# Rotigotine Effects on Early Morning Motor Function and Sleep in Parkinson's Disease: A Double-Blind, Randomized, pLacebo-Controlled Study (RECOVER)

**DOI:** 10.1002/mds.23441

**Published:** 2010-11-18

**Authors:** Claudia Trenkwalder, Bryan Kies, Monika Rudzinska, Jennifer Fine, Janos Nikl, Krystyna Honczarenko, Peter Dioszeghy, Dennis Hill, Tim Anderson, Vilho Myllyla, Jan Kassubek, Malcolm Steiger, Marco Zucconi, Eduardo Tolosa, Werner Poewe, Erwin Surmann, John Whitesides, Babak Boroojerdi, Kallol Ray Chaudhuri, the RECOVER Study Group

**Affiliations:** 1 Department of Clinical Neurophysiology, University of Goettingen and Paracelsus-Elena KlinikKassel, Germany; 2 Neurology Division, University of Cape TownSouth Africa; 3 Department of Neurology, Jagiellonian University Medical CollegeKrakow, Poland; 4 Constantiaberg MediClinicCape Town, South Africa; 5 Department of Neurology, Hospital of Zala CountyZalaegerszeg, Hungary; 6 Clinic of Neurology, Pomeranian Medical UniversitySzczecin, Poland; 7 Department of Neurology, County HospitalNyiregyhaza, Hungary; 8 Central Carolina Neurology and SleepSalisbury, North Carolina, USA; 9 Department of Medicine, University of Otago, Christchurch, and Van Der Veer InstituteChristchurch, New Zealand; 10 Department of Neurology, Oulu University HospitalOulu, Finland; 11 Neurologische Klinik, Universität UlmUlm, Germany; 12 The Walton Centre of Neurology and NeurosurgeryLiverpool, United Kingdom; 13 Centro per i Disturbi del Sonno, Instituto Scientifico H San RaffaeleMilan, Italy; 14 Neurology Service, Centro de Investigación Biomédica en Red sobre Enfermedades Neurodegenerativas (CIBERNED), Hospital Clínic, IDIBAPS, Universitat de BarcelonaSpain; 15 Department of Neurology, Medical University InnsbruckInnsbruck, Austria; 16 UCB Pharma GmbHMonheim, Germany; 17 Schwarz Biosciences Inc, a member of the UCB GroupRaleigh, NC, USA; 18 NPF Centre of Excellence, Kings College Hospital and University Hospital Lewisham, Kings College and Institute of PsychiatryLondon, United Kingdom

**Keywords:** dopamine agonist, rotigotine, transdermal, motor function, sleep, quality of life

## Abstract

In a multinational, double-blind, placebo-controlled trial (NCT00474058), 287 subjects with Parkinson's disease (PD) and unsatisfactory early-morning motor symptom control were randomized 2:1 to receive rotigotine (2–16 mg/24 hr [n = 190]) or placebo (n = 97). Treatment was titrated to optimal dose over 1–8 weeks with subsequent dose maintenance for 4 weeks. Early-morning motor function and nocturnal sleep disturbance were assessed as coprimary efficacy endpoints using the Unified Parkinson's Disease Rating Scale (UPDRS) Part III (Motor Examination) measured in the early morning prior to any medication intake and the modified Parkinson's Disease Sleep Scale (PDSS-2) (mean change from baseline to end of maintenance [EOM], last observation carried forward). At EOM, mean UPDRS Part III score had decreased by −7.0 points with rotigotine (from a baseline of 29.6 [standard deviation (SD) 12.3] and by −3.9 points with placebo (baseline 32.0 [13.3]). Mean PDSS-2 total score had decreased by −5.9 points with rotigotine (from a baseline of 19.3 [SD 9.3]) and by −1.9 points with placebo (baseline 20.5 [10.4]). This represented a significantly greater improvement with rotigotine compared with placebo on both the UPDRS Part III (treatment difference: −3.55 [95% confidence interval (CI) −5.37, −1.73]; *P* = 0.0002) and PDSS-2 (treatment difference: −4.26 [95% CI −6.08, −2.45]; *P* < 0.0001). The most frequently reported adverse events were nausea (placebo, 9%; rotigotine, 21%), application site reactions (placebo, 4%; rotigotine, 15%), and dizziness (placebo, 6%; rotigotine 10%). Twenty-four-hour transdermal delivery of rotigotine to PD patients with early-morning motor dysfunction resulted in significant benefits in control of both motor function and nocturnal sleep disturbances. © 2010 Movement Disorder Society

## INTRODUCTION

Parkinson's disease (PD) is characterized by motor symptoms including nocturnal and early morning dystonia and akinesia and is also associated with nonmotor symptoms, such as sleep disorders.^1^ These sleep disorders may be secondary to the patient's underlying disease pathology but may, in part, be a consequence of motor complications and other nonmotor symptoms (such as nocturnal pain, insomnia, and vivid dreams), or medication use. Although the importance of identifying and treating sleep disorders in PD is well recognized, few published trials have prospectively studied their management.^2^–^5^

Effective management of nocturnal and early morning PD symptoms may be achieved with either a long-acting treatment or by continuous administration of a short-acting treatment.^2^,^3^,^6^ Rotigotine is a non-ergoline dopamine agonist that is applied once daily using a transdermal patch, providing 24 hours of continuous drug delivery, and is generally well tolerated.^7^ Significant treatment benefits of rotigotine have been observed in both early and advanced PD and include improvements in activities of daily living and motor symptoms, clinically relevant reductions in “off” time, and reductions in concomitant l-dopa dose.^8^ Rotigotine's potential to improve early morning motor function and sleep-associated problems in PD was demonstrated recently by two large controlled studies^9^ that showed a reduction in the proportion of patients awakening in an “off” state.

This study compared the effects of rotigotine and placebo on early morning motor function and nocturnal sleep disturbances in subjects with PD. (It should be noted that while dopamine agonists have a variable effect on sleep architecture, this was not addressed in this study.) Effects on nocturnal symptoms such as limb restlessness, tremor, and cramps and on other nonmotor symptoms such as pain and mood were also investigated. To our knowledge, this is the first large, controlled trial in PD to use sleep outcomes as a coprimary outcome measure.

## METHODS

### Design

The RECOVER (Randomized Evaluation of the 24-hour Coverage: Efficacy of Rotigotine) study (NCT00474058) was a Phase 3b, double-blind, placebo-controlled, randomized, parallel-group, two-arm trial undertaken in 49 centers in 12 countries. Subjects were recruited between May 2, 2007 and November 17, 2008. Maximum study duration per subject was 22 weeks, with a maximum duration of treatment of 12 weeks.

### Subjects

Men and women (aged ≥18 years) with PD (Hoehn and Yahr Stage I-IV; both fluctuators and nonfluctuators) and unsatisfactory control of early morning motor symptoms as determined by the investigator (with the latter intended to reflect clinical practice), were eligible for inclusion. PD was defined by the presence of bradykinesia and at least one of the following: resting tremor, rigidity, or impairment of postural reflexes. Subjects not taking l-dopa were eligible for study inclusion as were those taking immediate-release l-dopa provided they had been on a stable dose for the 28 days prior to baseline (see Supporting Information text for study exclusion criteria).

Antiemetics without central dopaminergic activity were permitted to treat nausea and vomiting that occurred during study drug use. Anticholinergic agents, monoamine oxidase-B inhibitors, NMDA antagonists, entacapone, sedatives, hypnotics, selective serotonin reuptake inhibitors, anxiolytics, and other CNS medications were permitted if dose was stable for the 28 days prior to baseline and likely to remain so for the duration of the study. Controlled-release l-dopa, other centrally acting dopaminergic agents, monoamine oxidase-A inhibitors, tolcapone, budipine, or neuroleptics (except olanzapine, ziprasidone, aripiprazole, clozapine, or quetiapine) were prohibited from 28 days prior to baseline and during the study.

The study was conducted in accordance with Good Clinical Practice and the Declaration of Helsinki. The study protocol and amendments were approved by a national, regional, or Independent Ethics Committee or Institutional Review Board. All subjects provided written, informed consent before study participation.

### Protocol

Screening took place up to 4 weeks before the baseline evaluation on Day 1 when subjects were randomized 2:1 to receive rotigotine or placebo, stratified by site, using a computerized randomization schedule. Starting on Day 1 treatment was administered once daily in the morning using a 24-hour transdermal patch with identical-looking placebo patches to ensure blinding. Treatment was titrated to optimal dose (that at which investigator and subject felt that early morning motor impairment was adequately controlled) over 1–8 weeks, starting at 2 mg/24 hr and increasing in weekly increments of 2 mg/24 hr up to a maximum of 16 mg/24 hr. The dose was maintained at the optimal or maximal dose for a 4-week period (maintenance period) during which dose reduction (and alteration of concomitant l-dopa dose, if applicable) was not permitted. During the titration period, rotigotine dose could be back-titrated once if adverse events (AEs) occurred that were thought to be the result of excessive dopaminergic stimulation (see Supporting Information text). Subjects requiring back-titration immediately proceeded to the maintenance period.

Clinic visits took place at screening and baseline; every 2 weeks during dose titration; start and end of maintenance; and 30 days after treatment ended. Subjects were hospitalized for 2 nights at baseline (the first an adaptation night) and again at end of maintenance. Subjects who withdrew prematurely were asked to return to the clinic for a withdrawal visit. Efficacy assessments were performed after the first or second night of hospitalization depending on the measure (see Supporting Information text) and at the end of maintenance or the withdrawal visit.

### Outcome Measures

#### Coprimary Efficacy Measures

**FIG. 1:**
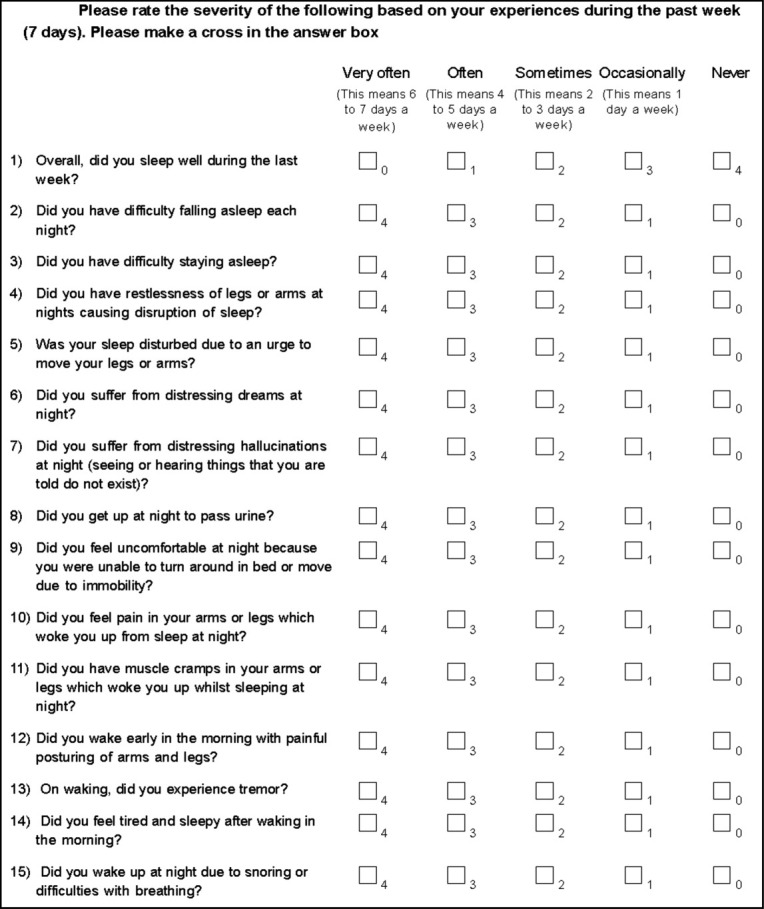
Modified Parkinson's Disease Sleep Scale (PDSS-2). ©Trenkwalder and Chaudhuri, 2010.

Motor function was assessed in the early morning, before new patch application or PD medication intake, using the Unified Parkinson's Disease Rating Scale (UPDRS) Part III (Motor Examination)^10^ while in the practically defined “off” condition. Sleep and nocturnal disability were assessed using a modified version of the original 15-item Parkinson's Disease Sleep Scale (PDSS-2; Fig. 1)^11^ on which individual items are scored from 0 to 4, with the total score ranging from 0 to 60 where higher scores indicate greater impairment.

#### Additional Efficacy Measures

The 15 individual items of the PDSS-2 were examined as post hoc efficacy measures. Three PDSS-2 domain scores were also calculated post hoc, by summing individual item scores in groups of five (for a maximum score of 20): “disturbed sleep” (items 1–3, 8, and 14); “motor symptoms at night” (items 4–6, 12, and 13); “PD symptoms at night” (items 7, 9–11, and 15).^11^

Secondary efficacy measures were the Nocturnal Akinesia, Dystonia and Cramps Score (NADCS),^12^ and number of nocturias. Exploratory outcome measures were the Parkinson's Disease Non-Motor Symptom scale (NMS)^13^; the Beck Depression Inventory (BDI-II)^14^; an 11-point Likert pain scale; the short-form Parkinson's Disease Questionnaire (PDQ-8)^15^; and the UPDRS Part II (activities of daily living) and UPDRS Part IV (complications of therapy) domains^10^ (see Supporting Information text).

#### Safety and Tolerability Measures

Safety and tolerability were assessed throughout the study and up to 30 days after treatment discontinuation by monitoring the frequency and severity of AEs (including clinically relevant laboratory parameter abnormalities) and any changes in vital signs, physical and neurological findings, and ECGs. Emergence of impulse control disorders was monitored using the modified Minnesota Impulsive Disorder Interview (mMIDI).^16^

### Statistical Analyses

A sample size of 336 subjects was required to detect a between-group difference of 3.5 points (standard deviation [SD] 9.3) in change from baseline UPDRS Part III score with a power of 90% at the 0.05 significance level using a two-sided *t* test. Unavoidable changes in the manufacturing process for rotigotine during the course of the trial led to recruitment being stopped to avoid enrolled subjects switching to patches from the new manufacturing process; this resulted in recruitment of fewer subjects than planned. Since the power remained >80% at the 0.05 significance level, the reduced sample size was deemed to have no impact on the validity of the trial.

Safety analyses were performed on all randomized subjects who received at least one dose of study drug. Efficacy analyses were performed on the full analysis set (FAS)—all randomized subjects who received at least one dose of study drug and had baseline and at least one post-baseline assessment for both coprimary efficacy measures (mean change in UPDRS Part III and PDSS-2 scores from baseline to end of maintenance [last observation carried forward (LOCF); i.e., modified intent-to-treat analysis]). The two coprimary endpoints were tested in a hierarchical sequential fashion, such that if the testing procedure demonstrated significance at the 5% level on UPDRS Part III, the test for the PDSS-2 score was performed (see Supporting Information text). This enabled evaluation of the motor and non-motor symptoms of PD without adjustments for multiple comparisons. To estimate treatment differences, analyses of covariance were performed with treatment and pooled site as factors and baseline score as the covariate.

## RESULTS

### Subject Disposition

**FIG. 2:**
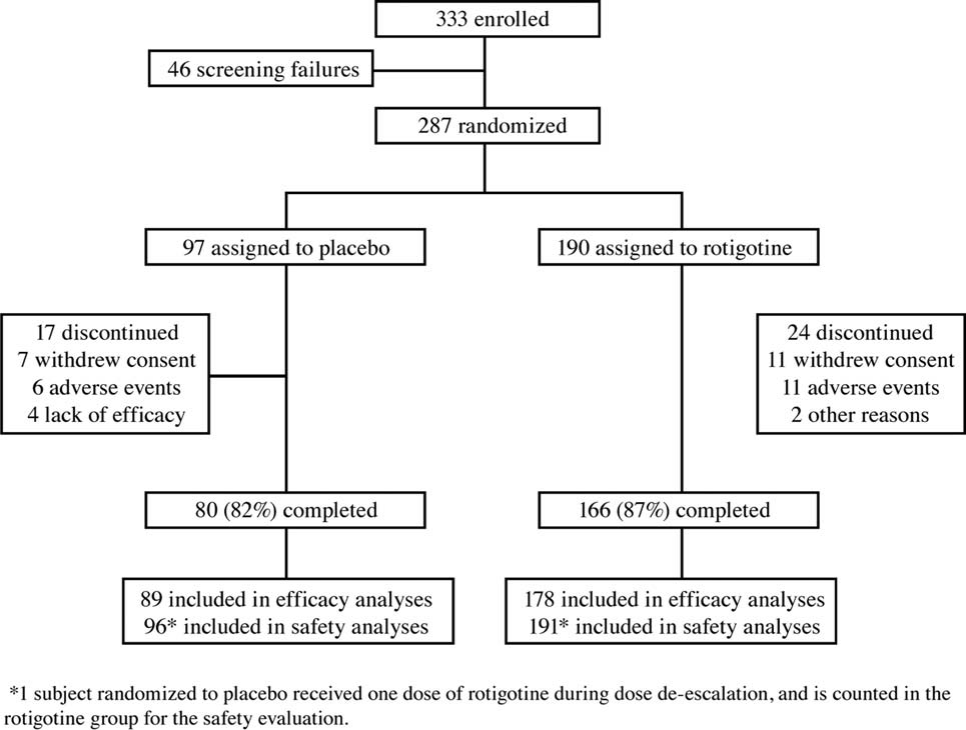
Subject disposition.

Of 333 subjects screened, 287 were randomized and 246 (86%) completed the study (Fig. 2). The most common reason for discontinuation was withdrawn consent. All 287 randomized subjects were evaluated for safety, while 267 subjects were included in the FAS and evaluated for efficacy.

### Baseline Characteristics

Baseline demographic and clinical characteristics, including UPDRS Part III and PDSS-2 total scores, were similar between treatment groups (Table 1). Most subjects (91%) had comorbid conditions at study entry, the most common of which were hypertension (44%), constipation (15%), depression (14%), and hypercholesterolemia (14%). Details of concomitant medication use are provided in the Supporting Information.

**TABLE 1:** Baseline demographic and clinical characteristics (safety population^a^)

	Placebo (n = 96)	Rotigotine (n = 191)
Age, mean (SD); range	64.4 (10.6); 37–86	64.8 (9.3); 37–85
Gender, n (%)		
Male	61 (64)	123 (64)
Female	35 (36)	68 (36)
BMI, mean (SD) kg/m^2^; range	26.6 (4.5); 16–41	26.6 (4.1); 16–43
Race, n (%)		
White or Caucasian	85 (89)	177 (93)
Black	1 (1)	2 (1)
Asian	1 (1)	1 (<1)
Other	9 (9)	11 (6)
L-DOPA use, n (%)		
No	17 (18)	36 (19)
Yes	79 (82)	155 (81)
Time since first diagnosis, mean (SD) years; range	4.9 (4.6); 0–26	4.6 (4.2); 0–23
Disease severity at baseline, UPDRS Part III sum score categories, n (%)		
0–9	2 (2)	5 (3)
10–19	12 (13)	38 (20)
20–29	32 (33)	60 (31)
30–39	22 (23)	49 (26)
≥40	28 (29)	38 (20)
UPDRS Part III mean (SD) score^b^	32.0 (13.3)	29.6 (12.3)
PDSS-2 total mean (SD) score; range^b^	20.5 (10.4); 3–49	19.3 (9.3); 1–49

a 1 subject randomized to placebo received one dose of rotigotine during dose de-escalation and is counted in the rotigotine group for the safety population.

b Rotigotine, n = 190.

### Coprimary Efficacy Outcomes

**FIG. 3:**
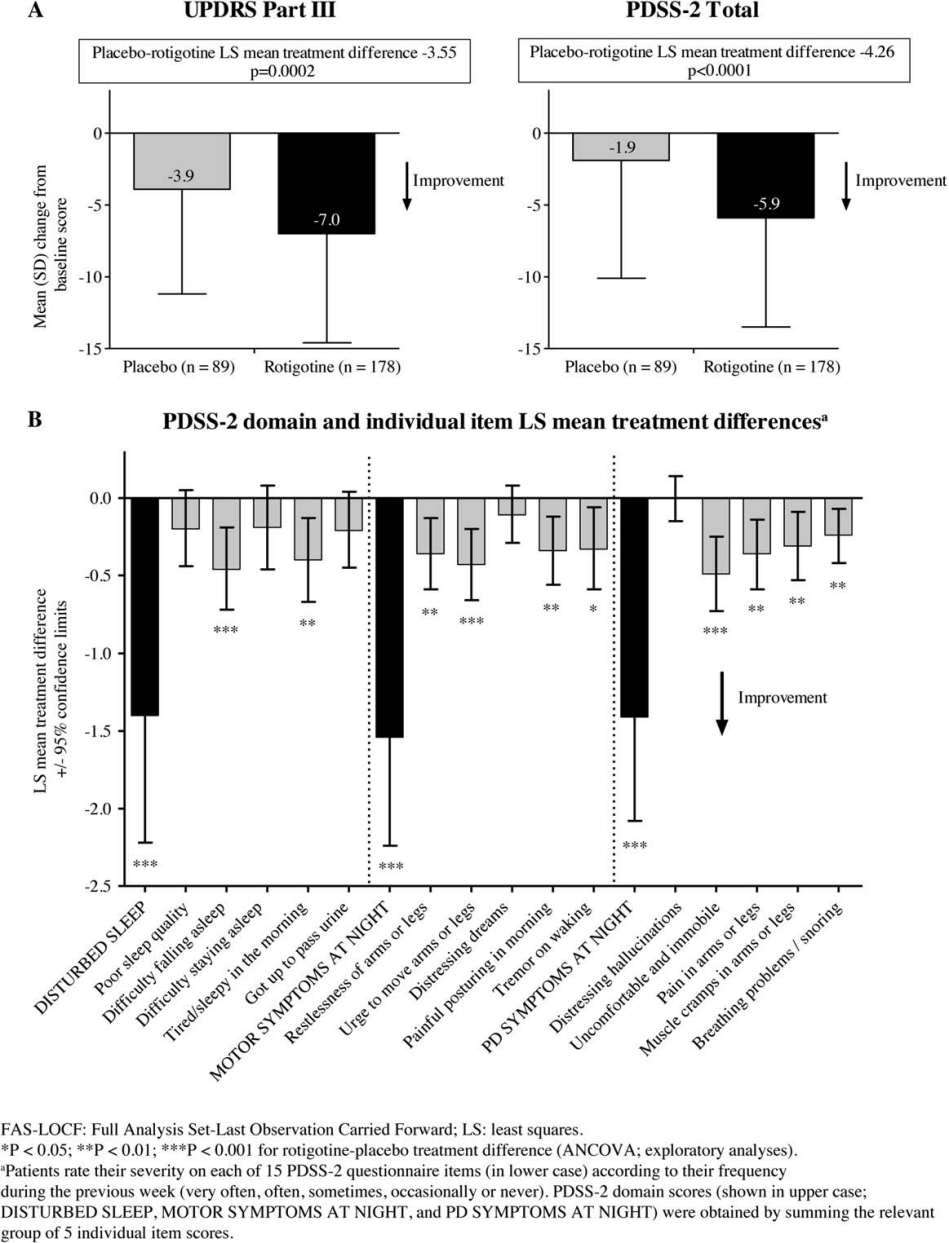
Mean change from baseline to end of maintenance in (**A**) UPDRS Part III scores and PDSS-2 total scores and (**B**) PDSS-2 domain and individual item LS mean rotigotine-placebo treatment differences (FAS-LOCF).

Mean UPDRS Part III scores measured in the early morning and PDSS-2 total scores showed significantly greater improvement with rotigotine than placebo from baseline to end of maintenance (least squares [LS] mean [95% confidence interval (CI)] treatment differences of −3.55 [−5.37, −1.73] (*P* = 0.0002) in UPDRS Part III and −4.26 [−6.08, −2.45] (*P* < 0.0001) in PDSS-2 [Fig. 3A]). These treatment differences were similar for l-dopa treated patients (−3.14 [−5.22, −1.05] in UPDRS Part III and −4.11 [−6.18, −2.05] in PDSS-2) and de novo patients (−4.84 [−8.32, −1.36] in UPDRS Part III and −4.54 [−7.94, −1.15] in PDSS-2).

### Additional Efficacy Outcomes

All three PDSS-2 domain scores showed significantly greater improvement with rotigotine than placebo from baseline to end of treatment (Fig. 3B). All 15 individual PDSS-2 items except “distressing hallucinations” showed improvement in the rotigotine group; with significant improvements on 10 items, particularly “difficulty falling asleep,” “urge to move arms or legs,” and “uncomfortable and immobile” (Fig. 3B). Likewise, greater improvement was seen with rotigotine than placebo on the NADCS (LS mean treatment difference −0.41 [−0.79, −0.04]; *P* = 0.030) (Table 2). The number of nocturias in each group changed by a mean of −0.3 from baseline, and no significant difference was seen between groups (Table 2; LS mean treatment difference −0.02 [−0.29, 0.25]; *P* = 0.88).

**TABLE 2:** Mean change from baseline to end of treatment in the secondary outcomes, NADCS and number of nocturias, and the exploratory outcomes, NMS total and individual domain scores, BDI-II, Likert pain scale, PDQ-8, UPDRS Part II (FAS-observed cases)

	Mean (SD) baseline score		Mean (SD) change	
		
	Placebo	Rotigotine	Placebo	Rotigotine
NADCS	2.7 (2.1) (n = 89)	2.9 (2.2) (n = 178)	−0.7 (2.1) (n = 89)	−1.2 (1.8)^a^ (n = 178)
Number of nocturias	2.0 (1.8) (n = 89)	1.9 (1.4) (n = 176)	−0.3 (1.6) (n = 89)	−0.3 (1.3) (n = 176)
NMS total score	41.3 (33.5) (n = 87)	41.1 (34.5) (n = 173)	−3.9 (25.5) (n = 86)	−10.3 (21.2)^a^ (n = 172)
Individual NMS domain				
Cardiovascular	1.1 (1.9) (n = 89)	1.0 (1.9) (n = 178)	−0.2 (1.7) (n = 88)	0.0 (1.9) (n = 178)
Sleep/fatigue	9.1 (8.6) (n = 89)	9.5 (9.4) (n = 176)	−1.5 (6.2) (n = 88)	−3.7 (6.8)^b^ (n = 176)
Mood/cognition	7.3 (10.0) (n = 89)	7.1 (9.3) (n = 178)	0.2 (11.1) (n = 88)	−3.0 (7.5)^c^ (n = 178)
Perception/hallucinations	0.4 (1.4) (n = 89)	0.5 (1.7) (n = 178)	0.0 (1.3) (n = 88)	0.1 (2.4) (n = 178)
Attention/memory	3.9 (5.0) (n = 89)	4.5 (6.4) (n = 178)	0.0 (4.8) (n = 88)	−0.3 (4.6) (n = 178)
Gastrointestinal tract	4.2 (5.7) (n = 89)	3.6 (4.4) (n = 178)	0.0 (3.9) (n = 88)	−0.6 (2.6) (n = 178)
Urinary	6.7 (7.5) (n = 89)	6.6 (7.5) (n = 176)	−0.7 (5.0) (n = 88)	−1.1 (4.9) (n = 176)
Sexual function	3.9 (6.3) (n = 87)	3.7 (5.7) (n = 177)	−0.4 (4.3) (n = 86)	−0.2 (4.9) (n = 177)
Miscellaneous	5.1 (5.6) (n = 89)	4.8 (6.4) (n = 178)	−1.0 (4.2) (n = 88)	−1.3 (3.5) (n = 177)
BDI-II	12.6 (8.8) (n = 89)	12.3 (8.0) (n = 178)	−0.8 (7.6) (n = 89)	−2.7 (5.7)^a^ (n = 177)
Likert pain scale	2.6 (2.5) (n = 89)	2.8 (2.4) (n = 178)	−0.1 (2.3) (n = 88)	−0.9 (2.2)^b^ (n = 178)
PDQ-8	31.1 (17.0) (n = 89)	30.8 (18.2) (n = 177)	−1.2 (13.7) (n = 89)	−6.9 (11.9)^c^ (n = 176)
UPDRS Part II	13.5 (6.3) (n = 89)	12.7 (5.6) (n = 178)	−1.3 (3.4) (n = 89)	−2.6 (3.6)^c^ (n = 178)

a *P* < 0.05.

b *P* < 0.01.

c *P* < 0.001 for rotigotine-placebo treatment difference (ANCOVA; Exploratory Analyses).

BDI-II, Beck depression inventory; PDQ-8, short-form Parkinson's Disease questionnaire; NADCS, Nocturnal Akinesia, Dystonia and Cramps Score; NMS, Parkinson's Disease Nonmotor Symptom Assessment Scale; UPDRS, Unified Parkinson's Disease Rating Scale; FAS, Full Analysis Set.

The mean NMS total score showed greater improvement with rotigotine than placebo from baseline to end of treatment (LS mean [95% CI] treatment difference −6.65 [−11.99, −1.31]; *P* = 0.015) (Table 2) with significant differences on the individual scores for sleep/fatigue and mood/cognition (LS mean treatment differences of −2.03 [−3.31, −0.75]; *P* = 0.002 and −3.40 [−5.22, −1.58]; *P* = 0.0003, respectively). Numerical differences in favor of rotigotine were seen for attention/memory, gastrointestinal tract, urinary, and miscellaneous (Table 2).

Greater improvements with rotigotine than placebo from baseline to end of treatment were seen on the BDI-II (LS mean treatment difference −2.01 [−3.55, −0.47]; *P* = 0.011); Likert pain scale (−0.77 [−1.28, −0.25]; *P* = 0.004); PDQ-8 (−5.74 [−8.74, −2.75]; *P* = 0.0002), and UPDRS Part II (−1.49 [−2.32, −0.65]; *P* = 0.0005) (Table 2). UPDRS Part IV individual item scores showed very little change from baseline to end of treatment in either group (see table in Supporting Information text), with no significant between-group differences.

Analysis of all outcome measures using observed cases, and a per protocol analysis of the two coprimary outcome measures yielded similar results to those reported above using LOCF.

### Safety and Tolerability Outcomes

The mean duration of drug exposure was 73 days in the placebo group and 71 days in the rotigotine group. During the titration phase, 7 (7%) placebo-treated and 40 (16%) rotigotine-treated subjects decreased their dose by 2 mg/24 hour (or placebo equivalent) due to tolerability concerns. At the start of maintenance, 67 (35%) rotigotine-treated subjects were taking the maximal dose of 16 mg/24 hour. Overall, 80% of subjects were compliant (placebo, 76%; rotigotine 83%).

The most frequently reported AEs during treatment are shown in Table 3. Two rotigotine-treated subjects experienced a sleep attack. One rotigotine-treated subject had positive findings of compulsive sexual behavior on the structured psychiatric interview, which was not reported as an impulse control disorder AE (this subject experienced one mild AE of appetite disorder/decreased appetite). A further 9 subjects (placebo, 2 [2%]; rotigotine, 7 [4%]) had a positive result on at least one mMIDI module.

**TABLE 3:** Most frequently reported^a^ treatment-emergent AEs during overall treatment (safety population^b^)

	Number (%) subjects	
	
Adverse event	Placebo (n = 96)	Rotigotine (n = 191)
At least 1 treatment-emergent AE^c^	54 (56)	137 (72)
Nausea	9 (9)	41 (21)
Application and instillation site reactions	4 (4)	29 (15)
Dizziness	6 (6)	20 (10)
Dyskinesia	4 (4)	15 (8)
Headache	5 (5)	13 (7)

a At least 5% in either group.

b One subject randomized to placebo received one dose of rotigotine during dose de-escalation and is counted in the rotigotine group for the safety evaluation.

c Subjects could report more than one type of AE.

Most reported AEs were mild or moderate in intensity (placebo, 96%; rotigotine, 97%). In both treatment groups 6% of subjects discontinued due to any AE. Five (3%) rotigotine-treated subjects discontinued due to application/instillation site reactions, none of which was considered serious. There was no clear relationship between rotigotine dose and application site reaction rates. Serious AEs were reported by 5 (5%) placebo-treated subjects and 10 (5%) rotigotine-treated subjects; only visual hallucination in 1 rotigotine-treated subject led to study withdrawal. Two subjects, both in the placebo group, died during the study (one committed suicide and the other died of pneumonia aspiration). There were no clinically relevant changes in vital signs or ECG findings, and few laboratory test values of clinical relevance.

## DISCUSSION

RECOVER is the first large-scale, double-blind, randomized trial to investigate early morning motor function and sleep as coprimary outcome measures in PD. In this study, 24-hour transdermal rotigotine treatment was associated with significant benefits versus placebo in the management of early morning motor impairment and nocturnal sleep disturbances. Rotigotine was also associated with improvements in night-time disabilities (such as limb restlessness, immobility, pain, and cramps), and possibly dopaminergic nonmotor daytime symptoms (such as fatigue and mood) as well.

Other dopamine agonists and continuous l-dopa/DDCI infusion have been used successfully for treating early morning motor disabilities.^3^,^17^,^18^ The treatment difference of 3.55 points on the UPDRS Part III seen in this 4-week study is consistent with treatment differences of 3.91 and 3.82 points on the UPDRS Part III at the end of 11 weeks of treatment with rotigotine at doses of 6 mg/24 hr and 8 mg/24 hr in an earlier dose-ranging study.^19^ Likewise, as for rotigotine in the current study, improvements from baseline of approximately 7 points on the UPDRS Part III have been described over periods of 6–24 weeks for cabergoline,^3^ l-dopa continuous infusion,^17^ and ropinirole controlled release.^18^

The clinimetrics of the PDSS are well established^21^ and validated in several studies across the world in independent populations.^22^–^25^ In this study, a modified version of the PDSS—the PDSS-2^11^—was used; this modified scale was developed to better reflect treatment effects on nocturnal disabilities and has been validated using patients' and caregivers' observations on nocturnal problems. The significant improvement in sleep seen on the PDSS-2 is consistent with results from a previous prospective open-label trial of rotigotine that used the original PDSS.^20^ Sleep disturbances in PD patients may be caused by a myriad of factors, including nocturnal motor problems, mood or pain, and dopaminergic AEs; likewise pain in PD may be the consequence of other PD symptoms, such as akinesia and rigidity. Musculoskeletal problems too might cause nocturnal pain. Improving nocturnal rigidity may benefit sleep and pain directly while better sleep continuity could be expected to improve pain in PD generally. Hence, in this study, the improvement in sleep produced by rotigotine may be the result of the improvement in nocturnal motor symptoms (restlessness of arms or legs, urge to move arms or legs, painful posturing in the morning, and tremor on awakening). Likewise, improvement on the individual PDSS-2 item—“painful posturing in the morning”—may reflect the overall early morning improvement in akinesia and dystonia observed on the UPDRS Part III: if akinesia and dystonia improve, pain is relieved and in turn an overall early morning benefit is seen. The reported improvements in depressive symptoms (BDI-II and NMS domain 2) and pain (Likert scale and PDSS-2 item 10) might also have contributed to the better sleep outcomes with rotigotine treatment. In contrast, number of nocturias showed little change during the study and is unlikely to have had any impact on sleep outcomes.

Subjective patient assessments in open-label investigations have suggested that long-acting dopaminergic drugs and formulations may improve sleep dysfunction in PD patients, possibly through improvement of sleep maintenance insomnia.^2^–^4^,^6^,^17^,^26^ Indeed, continuous delivery of rotigotine through the night could explain the reductions in nocturnal PD symptoms, and might also have counteracted any sleep problems caused by loss of l-dopa activity. Such observations have been reported previously with cabergoline and overnight apomorphine infusion.^4^,^27^

As treatment of the nonmotor symptoms of PD is a key unmet need in management of the disease as identified in recently published guidelines,^28^ an additional outcome in the current study was to investigate the nonmotor effects of rotigotine examined in pilot studies.^29^ Rotigotine treatment effects in the NMS domains of sleep/fatigue and mood/cognition support the findings from the PDSS-2 and the BDI-II. The treatment benefit seen in the mood/cognition domain is most likely due to an improvement in mood, as the BDI-II showed a significant effect of rotigotine on depressive symptoms.

Rotigotine was generally well tolerated in this study. The reported dopaminergic AEs are comparable with those seen with other nonergoline dopamine agonists.^30^ The occurrence of skin reactions showed no clear dose relationship and is related to use of the transdermal patch rather than to an allergic reaction to rotigotine. Few subjects showed emergence of impulsive behavior, with only one rotigotine-treated subject having a positive result on the structured psychiatric interview. Two rotigotine-treated subjects reported a sleep attack.

A significant strength of this study is the collection, under standardized conditions, of controlled data describing nocturnal disturbances in PD, including disease-specific problems such as nocturnal akinesia, restlessness, and nightmares. While this and other trials that examine early morning akinesia use data based on the previous night's sleep in a hospital environment, our study also used the PDSS-2, which collects data on the past week of sleep in the patient's own home. However, the lack of sleep laboratory data to measure sleep parameters and their changes objectively is a limitation. In addition, the inclusion of patients based on unsatisfactory control of early morning motor symptoms in the opinion of the investigator may have led to variation in the study population—that the majority (82%) of patients were taking l-dopa at study entry indicates the inclusion of a minority of de novo patients, which may have produced some heterogeneity in the observed treatment response.

Sleep disturbance; nocturnal limb restlessness; cramps, pain, and immobility; and impairment of early morning motor function, mood, and health-related quality of life are common and important nocturnal, early-morning, and daytime problems for PD patients. This study demonstrated significant treatment benefits with rotigotine on each of these outcomes. Indeed, this study is the first to confirm improvements in sleep-related PD problems with a dopaminergic treatment in a multicenter, double-blind, randomized, placebo-controlled trial using a patient-reported outcome as a coprimary outcome measure. Moreover, it has demonstrated the clinical benefits of continuous rotigotine delivery, as efficacy was maintained throughout the night and into the next morning, significantly improving the start of the new day, one of the most troubling times for PD patients. However, whether the effects of rotigotine on the nonmotor symptoms of PD are the result of its effects on motor symptoms, or whether there are additional effects of rotigotine which affect nonmotor qualities such as sleep fragmentation, dreaming, respiration and mood, which are unrelated to an improved motor condition, is still unclear and further objective study of sleep outcomes in PD patients may be warranted.

## Financial Disclosure:

K. Ray Chaudhuri, J. Kassubek, E. Tolosa, C. Trenkwalder, B. Kies, M. Steiger, and J. Fine have received honoraria and/or logistics reimbursement and/or consultancy fees from UCB or from Schwarz Pharma Ltd. In additon, C. Trenkwalder has received honoraria from UCB, GSK, Novartis, Mundipharma, TEVA, Cephalon, Desitin, and Boehringer Ingelheim, and has served on advisory boards for UCB, Boehringer Ingelheim, Solvay, and Cephalon. E. Tolosa has received honoraria from Novartis, Boehringer Ingelheim, UCB, Abbott, Teva, and Lundbeck as well as an educational grant from GSK. K. Ray Chaudhuri has received honoraria and consulting fees from Solvay, GSK, Boehringer Ingelheim, and UCB Pharma. B. Kies has received honoraria from Sanofi-Aventis and Novartis; and logistics reimbursement from Sanofi-Aventis and UCB. J. Fine has received clinical trial investigator fees from Merck Serona and Allergan and has worked as a medical advisor for Sanofi-Aventis. J. Kassubek has received advisory board fees and honoraria from UCB, GSK, and Boehringer Ingelheim and honoraria from Merz. W. Poewe has received consultancy and lecture fees from GSK, Boehringer Ingelheim, and UCB in relation to clinical drug development programmes for PD and RLS. J.C. Möller has received honoraria from AstraZeneca and MEGS and a grant from the Michael J. Fox Foundation. M. Steiger has received educational support from GSK, UCB Pharma, and Boehringer Ingelheim and honoraria from GSK, Teva, and Britannia Pharmaceuticals. In addition, he has acted as an advisor for Teva Pharmaceuticals, Solvay, Archimedes, MEDA Pharmaceuticals. T. Anderson has received grants from the Canterbury Medical Research Foundation, NZ, and the Neurological Foundation of New Zealand and honoraria from Boehringer Ingelheim. M. Rudzinska has received honoraria from GSK, UCB and Servier and K. Honczarenko from GSK. V. Myllylä has served on advisory boards for UCB Pharma and Boehringer Ingelheim. M. Onofrj has received consultancy fees from GSK, Novartis, Boehringer Ingelheim and Neuron SpA. M. Siddiqui has received honoraria from Teva, Novartis, GSK and Boehringer Ingelheim. E. Surmann, J. Whitesides, and B. Boroojerdi are employees of the study sponsor; J. Whitesides and B. Boroojerdi receive stock options from this employment. J. Nikl, P. Dioszeghy, D. Hill, M. Zucconi, J.M. Gómez-Arguelles, and M. Hayes have no additional conflicts of interest to declare.

**Author's Roles:** Claudia Trenkwalder, Eduardo Tolosa, John Whitesides, Babak Boroojerdi, and K. Ray Chaudhuri were involved in the conception and design of this study. Claudia Trenkwalder, Bryan Kies, Monika Rudzinska, Jennifer Fine, Janos Nikl, Krystyna Honczarenko, Peter Dioszeghy, Dennis Hill, Tim Anderson, Vilho Myllyla, Jan Kassubek, Malcolm Steiger, Marco Zucconi, Eduardo Tolosa, Werner Poewe, K. Ray Chaudhuri, Marco Onofrj, Michael William Hayes, Mustafa Saad Siddiqui, Jens Carsten Möller, and Jose Maria Gómez-Arguelles were responsible for the acquisition of data, in the role of study investigators. Claudia Trenkwalder, Eduardo Tolosa, Erwin Surmann, John Whitesides, and Babak Boroojerdi conducted data analysis and interpretation. All authors were involved in drafting or critically revising the article for important intellectual content and for the final approval of the published manuscript.

## APPENDIX

### RECOVER Study Group

Marco Onofrj, MD, Universita G. D'Annunzio di Chieti-Pescara, Italy; Michael William Hayes FRACP, Concord Hospital, Concord, NSW, Australia; Mustafa Saad Siddiqui, MD, Wake Forest University School of Medicine, Winston-Salem, NC, USA; Jens Carsten Möller, MD, Philipps-University, Marburg, Germany; and Jose Maria Gómez-Arguelles, MD, Sanatorio Nuestra Señora del Rosario, Madrid, Spain.
