# Supplementary material for: Rotigotine Effects on Early Morning Motor Function and Sleep in Parkinson's Disease: A Double-Blind, Randomized, pLacebo-Controlled Study (RECOVER)
Source: Mov Disord. 2010 Nov 18;26(1):90–9. doi: 10.1002/mds.23441 (PMC3072524; doi:10.1002/mds.23441)

## Proposed online-only text

This trial was undertaken in Australia, Austria, Finland, Germany, Hungary, Italy, New Zealand, Poland, South Africa, Spain, UK, and USA. Women of child-bearing potential could participate in the study provided they were using adequate birth control methods and were not pregnant or nursing. Subjects were excluded if they had: a previous diagnosis of clinically relevant narcolepsy; sleep apnoea; REM sleep behavior disorder; periodic limb movement disorder or restless legs syndrome; evidence of an impulse control disorder (assessed by modified Minnesota Impulsive Disorder interview [mMIDI]);^16^ dementia; psychosis; or hallucinations (except where caused by antiparkinsonian medication use); current or past atopic eczema; or known hypersensitivity to the trial medication; clinically relevant hepatic or renal dysfunction; clinically relevant cardiovascular disorders (including QTcB interval of ≥500 ms or recent myocardial infarction); a seizure or stroke within the past year; neoplastic disease requiring treatment within the past year; recent history of symptomatic orthostatic hypotension; current or past atopic eczema; or a recent history of drug or alcohol abuse. Subjects were not eligible for study entry if they had previously experienced treatment failure with dopamine agonists (including rotigotine), had previously participated in a rotigotine clinical trial, had participated in another clinical trial within the past 28 days, or were currently participating in another clinical trial.

After completion of the 4-week maintenance period, drug dose (rotigotine or matched placebo) was gradually reduced in steps of 2 mg/24 hour every 2 days. Compliance was monitored by assessing unused supplies in returned medication packaging. Subjects who took 85–115% of their assigned dose were considered compliant.

The Parkinson’s Disease Non-Motor Symptom scale (NMS) has been validated in two major international studies involving over 700 patients, and is a clinician-rated scale designed to assess non-motor PD symptoms in nine different domains.^13^ Symptom severity is rated from 0 (“none”) to 3 (“severe”) while symptom frequency is rated from 1 (“rarely”) to 4 (“very frequent”). Total and individual NMS domain scores were calculated. The Beck Depression Inventory second edition (BDI-II) provides a quantitative measure for depression.^14^ It contains 21 items, each with a score from 0 to 3; scores range from 0 (least severe depression) to 63 (most severe depression). The 11-point Likert pain scale is a subject-rated scale in which average pain over the past 12 hours is rated on a scale of 0 (“no pain”) to 10 (“worst pain ever experienced”). The Short-form Parkinson’s Disease Questionnaire (PDQ-8) is a self-administered eight-item questionnaire that assesses various problems associated with PD.^15^ Subjects are asked to rate how often during the past month (“never”, “occasionally”, “sometimes”, “often”, “always”/“cannot do at all”) they had difficulty getting around in public; had difficulty dressing; felt depressed; had problems with close personal relationships; had problems with concentration; felt unable to communicate properly; had painful muscle cramps or spasms; felt embarrassed in public due to PD. PDQ-8 scores range from 0 (least impairment) to 32 (most impairment). The Nocturnal Akinesia, Dystonia and Cramps Score (NADCS) is an investigator-administered, subject-rated scale used to evaluate motor performance (nocturnal akinesia score) and sleep (dystonia and cramps scores). The severity of each symptom is rated on a scale of 0 (“normal”) to 4 (“maximal severity”).^12^

The UPDRS Part II (activities of daily living) domain is a 13-item assessment of speech, salivation, swallowing, handwriting, cutting food, and handling utensils, dressing, hygiene, turning in bed and adjusting clothes, falling (unrelated to freezing), freezing when walking, walking, tremor, and sensory complaints related to Parkinsonism. Each item is rated from 0 to 4 and scores range from 0 (no impairment) to 52 (worst impairment).^10^ The UPDRS Part IV (complications of therapy) domain is an 11-item assessment of treatment-related adverse effects over the past week. It includes dyskinesias (duration; disability; painful; early morning dystonia), clinical fluctuations (predictable “off” periods; unpredictable “off” periods; sudden onset of “off” periods; duration of “off” periods), other fluctuations (anorexia, nausea, or vomiting; sleep disturbance; symptomatic orthostasis). Scores range from 0 (fewest complications) to 23 (most complications).^10^

Efficacy assessments were performed at baseline and end of maintenance, after the first night of hospitalization (NMS, BDI-II, PDQ-8, number of nocturias) or after the second night of hospitalization before new patch application in the morning (UPDRS Parts II, III, IV; PDSS-2; Likert pain scale; NADCS). If a withdrawal visit took place within 24 hours of patch removal, efficacy data were considered to be the last valid observation and were carried forward. Exploratory efficacy endpoints were the mean change in secondary efficacy measures from baseline to end of treatment. “End of treatment” combined observed cases data from end of maintenance visits with data from all withdrawal visits, to ensure the assumptions for the primary endpoints were also true for the additional efficacy endpoints.

Almost all randomized subjects (placebo, 96%; rotigotine, 98%) took at least one concomitant medication during the study. Other than L-DOPA, the most frequently taken medications were agents acting on the renin-angiotensin system (placebo, 31%; rotigotine, 26%) and antithrombotic agents (placebo, 28%; rotigotine, 25%). Similar proportions of subjects in each group started L-DOPA treatment during the study (placebo, 8%; rotigotine, 9%).

**Supplemental table.** *Mean change from baseline to end of treatment in UPDRS Part IV individual item scores (FAS-observed cases)*

|  | **Mean (SD) baseline score** | | **Mean (SD) change** | |
| --- | --- | --- | --- | --- |
|  | **Placebo** | **Rotigotine** | **Placebo** | **Rotigotine** |
| Dyskinesia duration, on | 0.3 (0.5) (n = 89) | 0.4 (0.6) (n = 178) | 0.0 (0.5) (n = 89) | –0.1 (0.4) (n = 178) |
| Dyskinesia disability, on | 0.2 (0.5) (n = 88) | 0.2 (0.5) (n = 178) | 0.0 (0.5) (n = 88) | 0.0 (0.4) (n = 178) |
| Dyskinesia pain, on | 0.2 (0.6) (n = 88) | 0.2 (0.6) (n = 178) | 0.0 (0.4) (n = 88) | –0.1 (0.6) (n = 178) |
| Early morning dystonia | 0.2 (0.4) (n = 89) | 0.3 (0.5)  (n = 178) | -0.1 (0.4)  (n = 89) | –0.1 (0.4)  (n = 178) |
| Offs, predictable | 0.5 (0.5)  (n = 89) | 0.6 (0.5)  (n = 177) | 0.0 (0.4)  (n = 89) | 0.0 (0.4)  (n = 177) |
| Offs, unpredictable | 0.2 (0.4)  (n = 89) | 0.3 (0.4)  (n = 177) | 0.0 (0.4)  (n = 89) | 0.0 (0.4)  (n = 177) |
| Offs, period comes suddenly | 0.2 (0.4)  (n = 89) | 0.2 (0.4)  (n = 177) | 0.0 (0.2)  (n = 89) | 0.0 (0.3)  (n = 177) |
| Offs, periods of waking day | 1.0 (1.0)  (n = 89) | 1.0 (0.8)  (n = 178) | –0.1 (0.6)  (n = 89) | –0.3 (0.6)  (n = 178) |
| Anorexia, nausea, vomiting, on | 0.1 (0.2) (n = 89) | 0.1 (0.3) (n = 178) | 0.0 (0.4)  (n = 89) | 0.0 (0.3)  (n = 178) |
| Sleep disturbance, on | 0.5 (0.5) (n = 89) | 0.4 (0.5) (n = 178) | –0.1 (0.5)  (n = 89) | –0.1 (0.5)  (n = 178) |
| Symptomatic orthostasis, on | 0.1 (0.2)  (n = 89) | 0.1 (0.3)  (n = 178) | 0.0 (0.3)  (n = 89) | 0.0 (0.2)  (n = 178) |

UPDRS: Unified Parkinson’s Disease Rating Scale; FAS: Full Analysis Set.

## Supplemental Figure. *Rotigotine titration scheme*

##
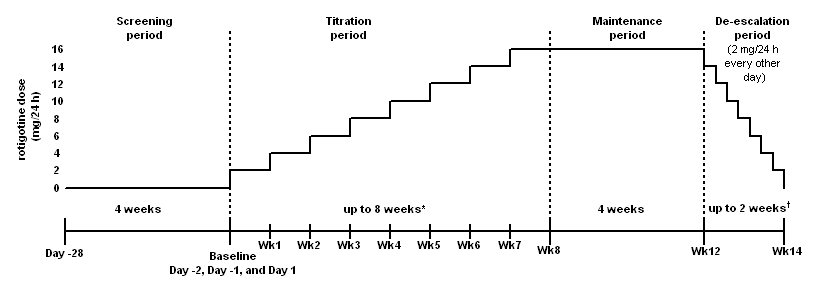

Supplement: Supplementary file 1 [file mds0026-0090-SD1.doc]
